# Supplementary material for: Regulation of eDHFR-tagged proteins with trimethoprim PROTACs
Source: Nat Commun. 2023 Nov 3;14:7071. doi: 10.1038/s41467-023-42820-3 (PMC10624689; doi:10.1038/s41467-023-42820-3)
Supplement: Supplementary file 1 — Supplementary Information [file 41467_2023_42820_MOESM1_ESM.pdf]

Regulation of eDHFR-tagged proteins with trimethoprim PROTACs

Jean M. Etersque[1†], Iris K. Lee[1†], Nitika Sharma[1†], Kexiang Xu[1], Andrew Ruff[1], Justin D. Northrup[1], Swarbhanu Sarkar[1], Tommy Nguyen[1], Richard Lauman[2], George M. Burslem[2,3], and Mark A. Sellmyer[1,2]*

1. Department of Radiology, Perelman School of Medicine, University of Pennsylvania, Philadelphia, PA, USA. 2.The Department of Biochemistry and Biophysics, Perelman School of Medicine, University of Pennsylvania, Philadelphia, PA, USA. 3. The Department of Cancer Biology, Perelman School of Medicine, University of Pennsylvania, Philadelphia, PA, USA.

[†]Authors contributed equally to this work, order was assigned alphabetically.

* Correspondence should be addressed to:
Mark A. Sellmyer (mark.sellmyer@pennmedicine.upenn.edu)
https://orcid.org/0000-0002-1407-1905

Department of Radiology
Perelman School of Medicine at the University of Pennsylvania
813A Stellar-Chance Labs
422 Curie Boulevard
Philadelphia, PA 19104-6059
Phone: 215-573-3212

A.

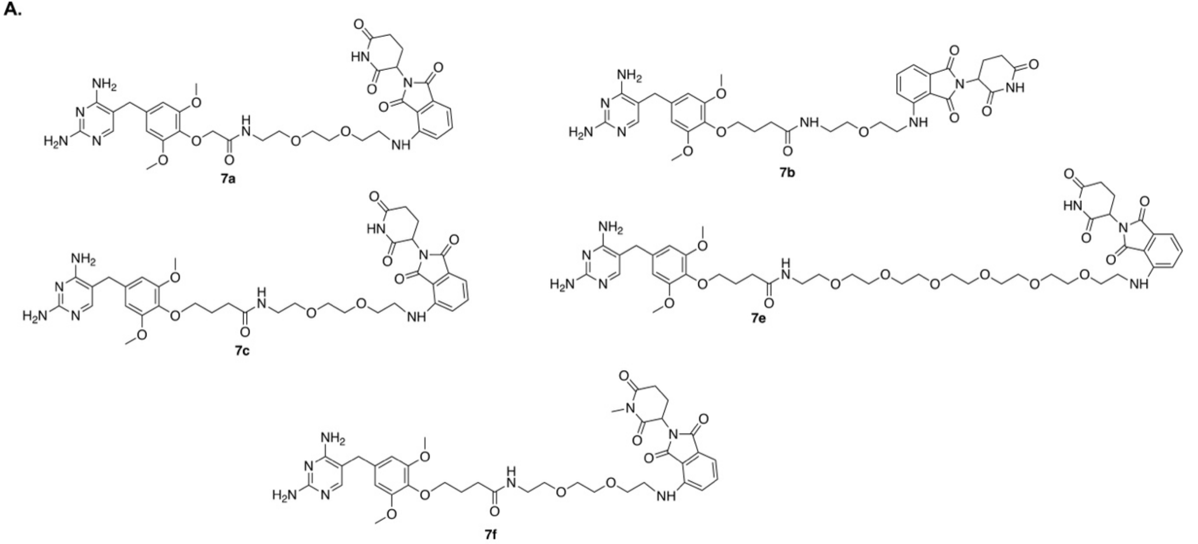

Supplementary Figure 1. Library of TMP PROTACs. Structures of the synthesized PROTACs **7a-c**, **e**, and **f**.

A.

IV (1mg/kg)

PK parameters	Unit	Mouse 1	Mouse 2	Mouse 3	Mouse 10	Mouse 11	Mean	SD	CV(%)
Cl_obs	mL/min/kg	59.8	233	56.6	125	210	137	83	60.3
$T_{1/2}$	h	0.666	0.361	0.707	0.177	0.518	0.486	0.220	45.2
C_0	ng/mL	2404	563	2792	1699	733	1638	987	60.3
AUC_{last}	h*ng/mL	278	70.8	294	133	77.9	171	108	63.3
AUC_{Inf}	h*ng/mL	279	71.4	294	134	79.3	172	108	62.9
$AUC_{_\%Extrap_}$obs	%	0.245	0.774	0.239	0.549	1.82	0.73	0.65	89.8
$MRT_{Inf_}$obs	h	0.231	0.220	0.207	0.105	0.240	0.201	0.055	27.3
AUC_{last}/D	h*mg/mL	278	70.8	294	133	77.9	171	108	63.3
$V_{ss_}$obs	L/kg	0.827	3.09	0.704	0.787	3.03	1.69	1.25	74.3

B

IP (50mg/kg)

PK parameters	Unit	Mouse 7	Mouse 8	Mouse 9	Mean	SD	CV(%)
$T_{1/2}$	h	9.56	0.925	2.29	4.26	4.64	109
T_{max}	h	0.250	0.500	0.0830	0.278	0.210	75.6
C_{max}	ng/mL	7730	8560	2830	6373	3097	48.6
AUC_{last}	h*ng/mL	8345	9100	4716	7387	2344	31.7
AUC_{Inf}	h*ng/mL	8502	9108	4720	7443	2378	31.9
$AUC_{_\%Extrap_}$obs	%	1.85	0.0829	0.0853	0.67	1.02	152
$MRT_{Inf_}$obs	h	2.09	0.812	1.71	1.54	0.66	42.7
AUC_{last}/D	h*mg/mL	167	182	94.3	148	47	31.7
F	%	99.1	106	55.0	87	28	31.9

C.

PO (10mg/kg)

PK parameters	Unit	Mouse 4	Mouse 5	Mouse 6	Mean	SD	CV(%)
$T_{1/2}$	h	NA	2.90	NA	NA	NA	NA
T_{max}	h	NA	0.0830	1.00	0.54	NA	NA
C_{max}	ng/mL	NA	1.12	2.48	1.80	NA	NA
AUC_{last}	h*ng/mL	NA	1.55	1.10	1.33	NA	NA
AUC_{Inf}	h*ng/mL	NA	3.66	NA	NA	NA	NA
$AUC_{_\%Extrap_}$obs	%	NA	57.5	NA	NA	NA	NA
$MRT_{Inf_}$obs	h	NA	3.98	NA	NA	NA	NA
AUC_{last}/D	h*mg/mL	NA	0.155	0.110	0.133	NA	NA
F	%	NA	0.0911	0.0645	0.0778	NA	NA

D.

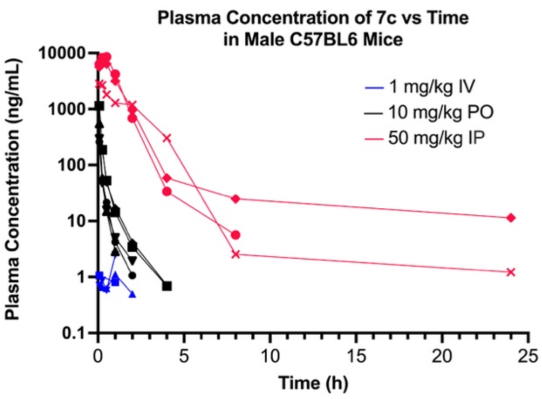

Supplementary Figure 2. ***In vivo*** **pharmacokinetic profile of 7c in male C57BL6 mice.**

A. Summary of **7c** IV pharmacokinetics (1 mg/kg) in mice. **B.** Summary of **7c** IP pharmacokinetics (50 mg/kg) in mice. **C.** Summary of **7c** PO pharmacokinetics (10 mg/kg) in mice. **D.** Mean plasma concentration vs. time graph by the delivery route (IV, IP, and PO) in mice.

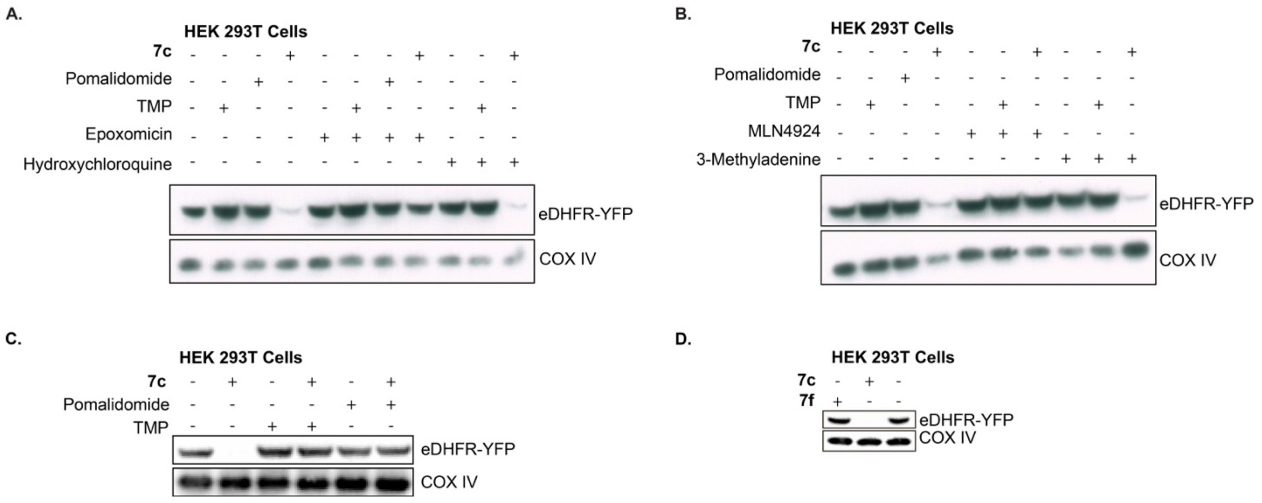

Supplementary Figure 3. Characterization of 7c-induced degradation mechanism of eDHFR fusion proteins. A. HEK293T-eDHFR-YFP cells were pre-incubated with 500 nM epoxomicin or 25 µM hydroxychloroquine sulfate for 1 hour, followed by additional 12 hours of incubation in presence of 100 nM **7c**, 25 µM TMP, or 2.5 µM pomalidomide. eDHFR-YFP was detected with anti-GFP antibody. eDHFR-YFP protein fusion is 45 kDa. COX IV = loading control, 18 kDa. n=3. **B.** HEK293T-eDHFR-YFP cells were pre-incubated with 500 nM MLN4924 or 25 µM 3-methyladenine for 1 hour followed by additional 12 hours of incubation in presence of 100 nM **7c**, 25 µM TMP, or 2.5 µM pomalidomide. eDHFR-YFP was detected with anti-GFP antibody. eDHFR-YFP protein fusion is 45 kDa. COX IV = loading control, 18 kDa. n=3 **C.** HEK293T-eDHFR-YFP cells were co-treated with 25 µM TMP or 2.5 µM pomalidomide and along with 100 nM **7c**. eDHFR-YFP was detected with anti-GFP antibody. eDHFR-YFP protein fusion is 45 kDa. COX IV = loading control, 18 kDa. n=2. **D.** HEK293T-eDHFR-YFP cells were incubated with 100 nM **7f**, 100 nM **7c**, or 500 nM epoxomicin for 24 hours. eDHFR-YFP was detected with anti-GFP antibody. eDHFR-YFP protein fusion is 45 kDa. COX IV = loading control, 18 kDa. n=3.

A.

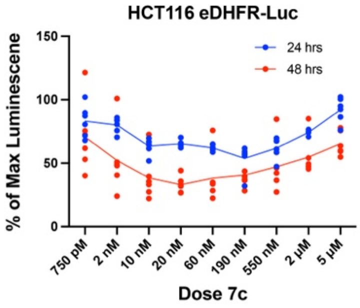

Supplementary Figure 4. Measuring degradation using 7c in HCT116 cells that have low Cereblon expression. HCT116 cells expressing eDHFR-Luciferase were incubated with **7c** for 24 and 48 hours. Following incubation, D-Luciferin was added to the cells and the luminescence was measured using a plate reader to assess the level of Luciferase expression. n=3, data points are mean ± SD from representative experiment, n=6 technical replicates.

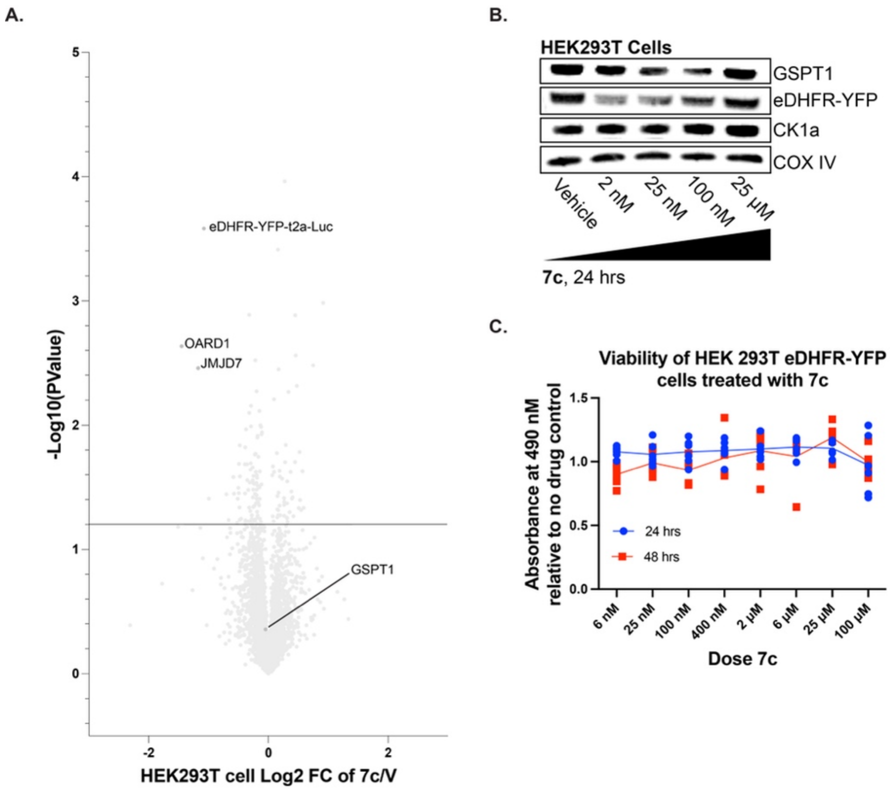

Supplementary Figure 5. Characterization of IMiD-sensitive proteins in HEK293T-eDHFR-YFP cells and cell viability. A. Mass spectrometry proteomics of cell lysates from HEK29T-eDHFR-YFP cells treated with 100 nM **7c** for 48 hours. Volcano plot shows effect of **7c** on protein levels in HEK-eDHRP-YFP cells relative to vehicle control. n=2, each experiment with 4 technical replicates, data point represents mean value. Statistical significance was determined using a two-tailed students t-test. **B.** Dose-response characterization of off-target binding and degradation of GSPT1 and CK1α in HEK293T-eDHFR-YFP cells after 24h incubation. GSPT1 detected with anti-eRF3 antibody, 56 kDa, eDHFR-YFP with anti-GFP antibody, 45 kDa, and CK1α with anti- CK1α antibody, 39 kDa. COX IV = loading control, 18 kDa. n=3. **C.** Dose response-cell viability characterization of HEK-eDHFR-YFP cells when treated with **7c** for either

24 or 48 hours compared to no drug control. n=1, data points are mean ± SD, n=6 technical replicates.

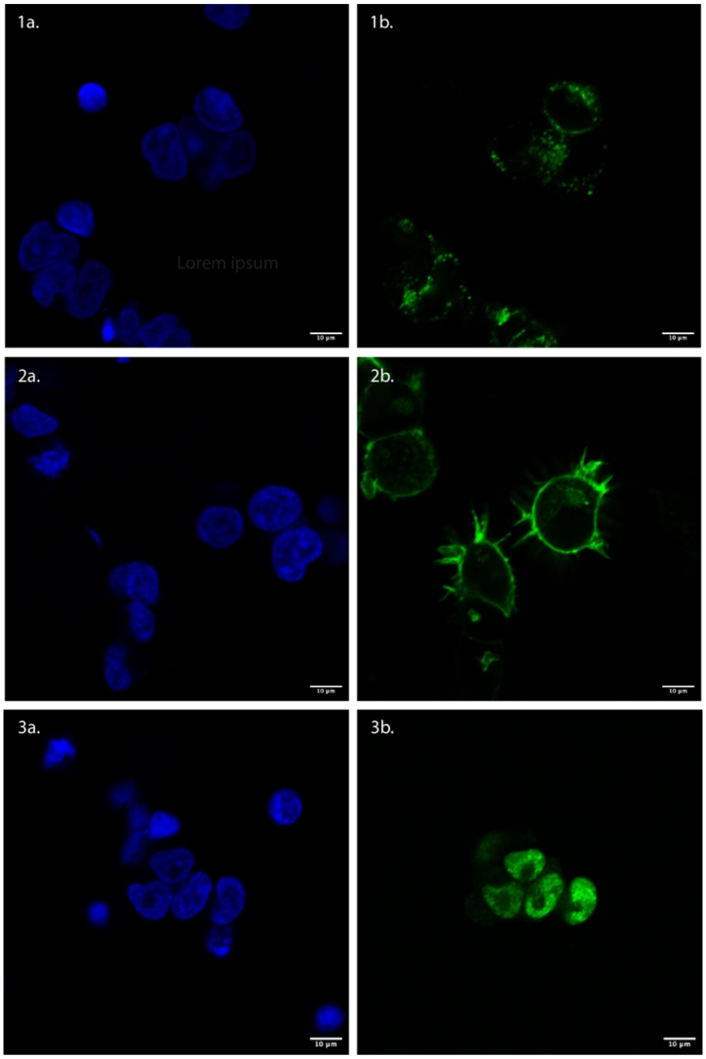

Supplementary Figure 6. Immunofluorescence microscopy of OVCAR8-eDHFR-POI-FLAG cells. Immunofluorescence of OVCAR8 cells expressing POI-eDHFR-FLAG constructs with AlexaFluor-488 secondary antibody against Anti-FLAG primary antibody (green), and DAPI nuclear staining (blue). 1a. IL2RB-eDHFR-FLAG DAPI-stained nucleus. 1b. CD122-eDHFR-FLAG localized in endosomes with AlexaFluor-488 secondary antibody 2a. LCK-eDHFR-FLAG DAPI-stained nucleus. 2b. Membrane associated LCK-eDHFR-FLAG with AlexaFluor-488

secondary antibody. 3a. RUNX1-eDHFR-FLAG DAPI-stained nucleus. 3b. RUNX1-eDHFR-FLAG localized in the nucleus with AlexaFluor-488 secondary antibody.

A.

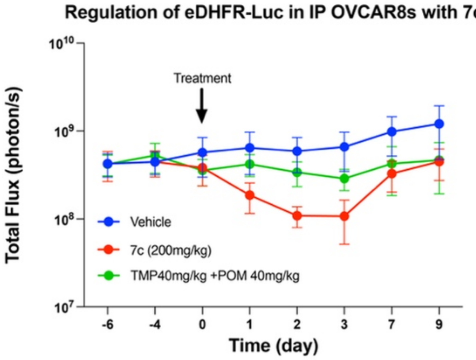

Supplementary Figure 7. Regulation of eDHFR-luciferase expression *in vivo* with TMP + pomalidomide admixture control. A. $10x10^6$ OVCAR8-DL cells were injected intraperitoneally (IP) in CD-1 nu/nu mice. Following 4 weeks of tumor growth, bioluminescence imaging (BLI) was performed to measure baseline luciferase expression (Day -4). On Day 0, 200 mg/kg of **7c**, 40 mg/kg TMP + 40 mg/kg pomalidomide admixture or vehicle was administered IP 3 times every 3 hours, and bioluminescence imaging (BLI) was performed1 hour after the last dose and monitored for 9 days. n=5 for each group, data points are mean ± SEM.

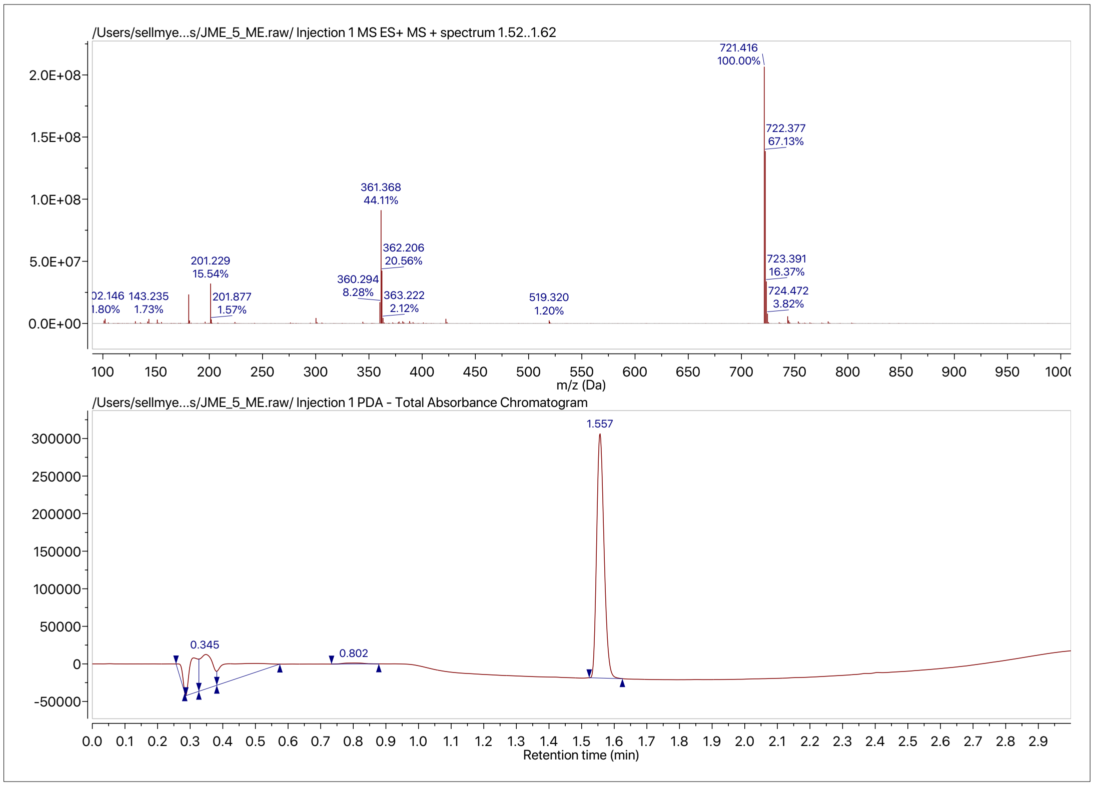

Supplementary Figure 8. LCMS spectrum of compound **7a**.

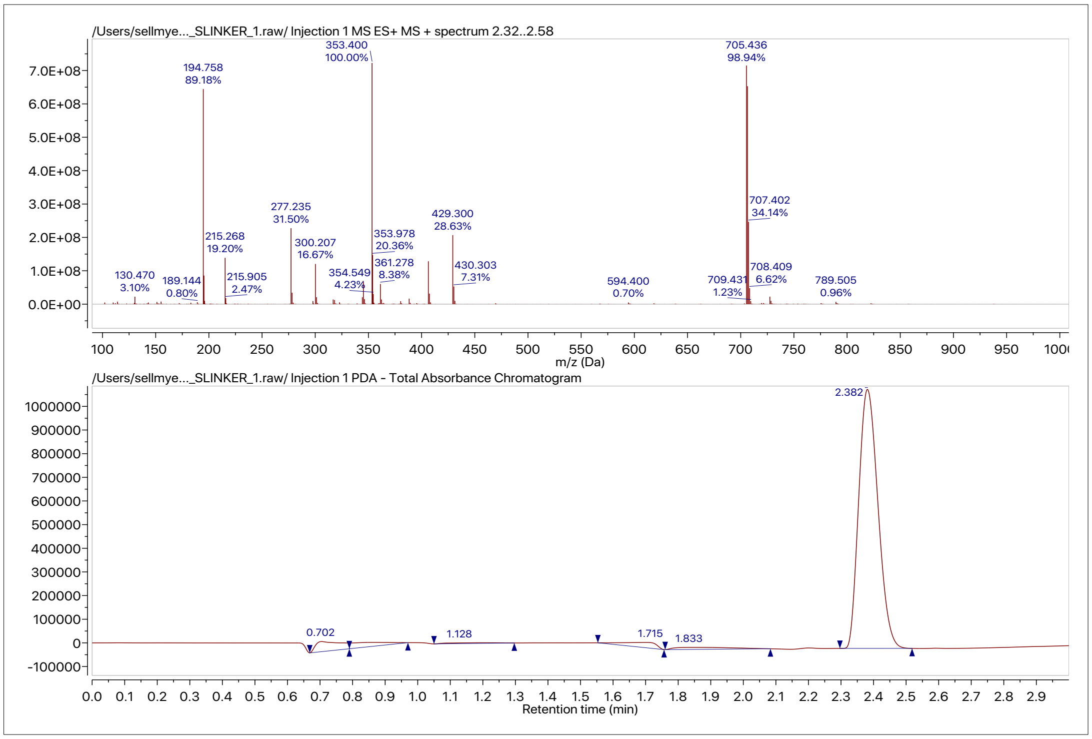

Supplementary Figure 9. LCMS spectrum of compound **7b**.

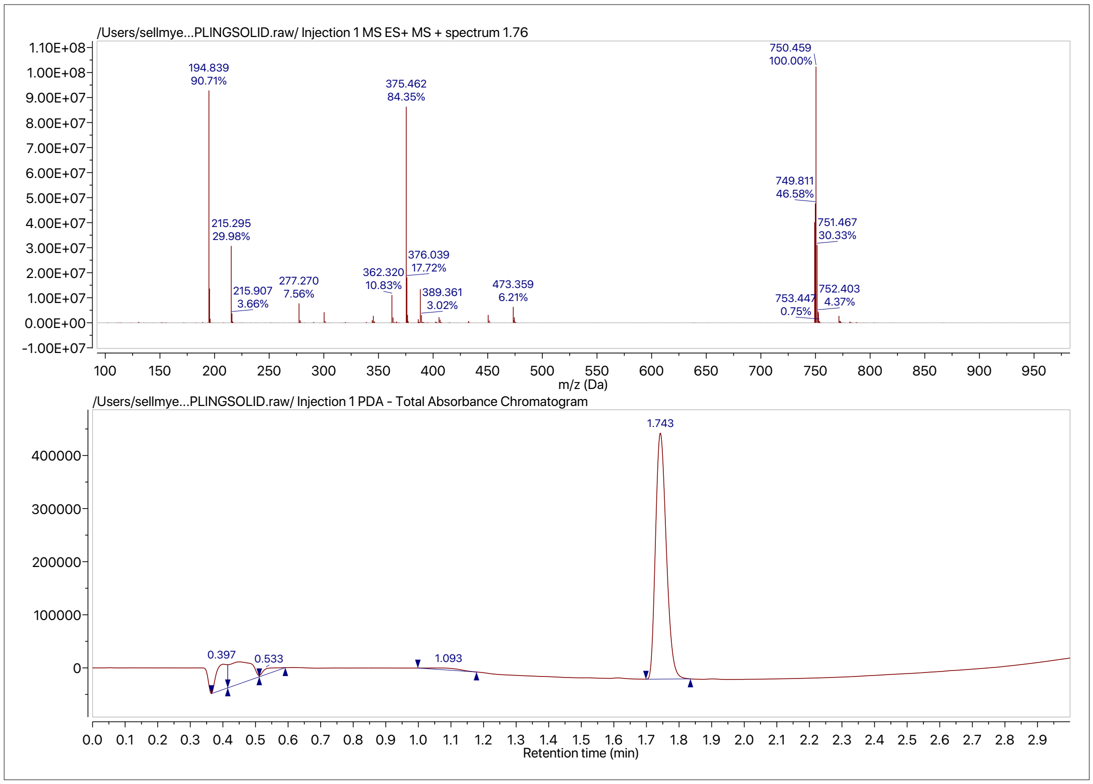

Supplementary Figure 10. LCMS spectrum **7c**.

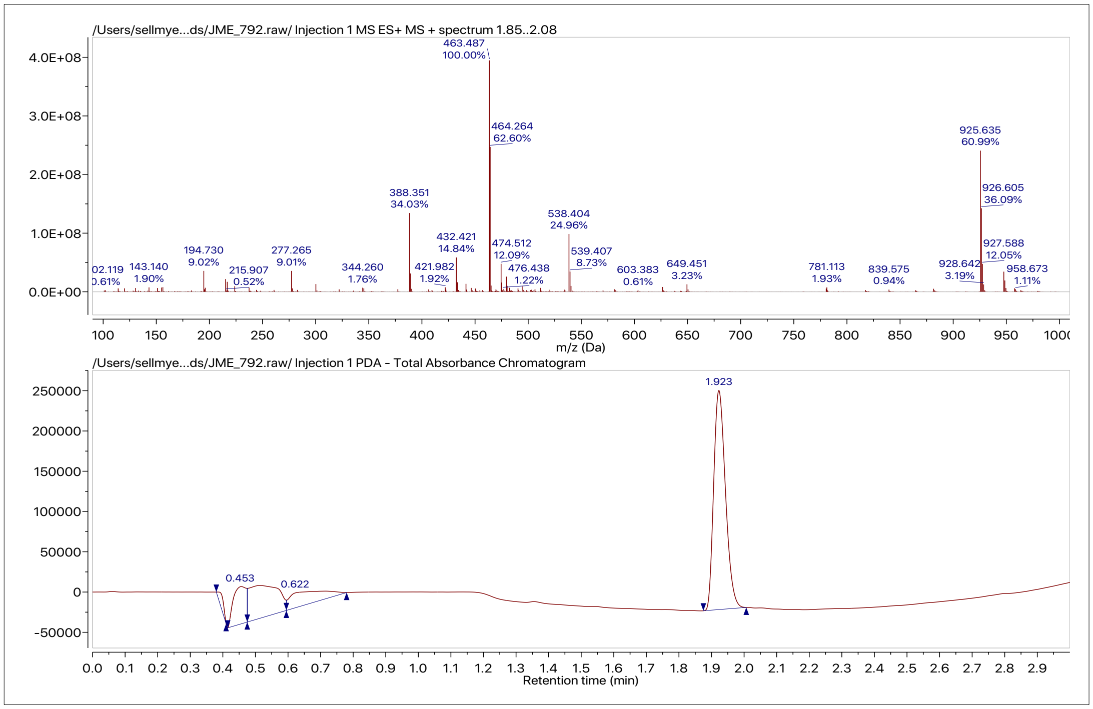

Supplementary Figure 11. LCMS spectrum of compound **7e**.

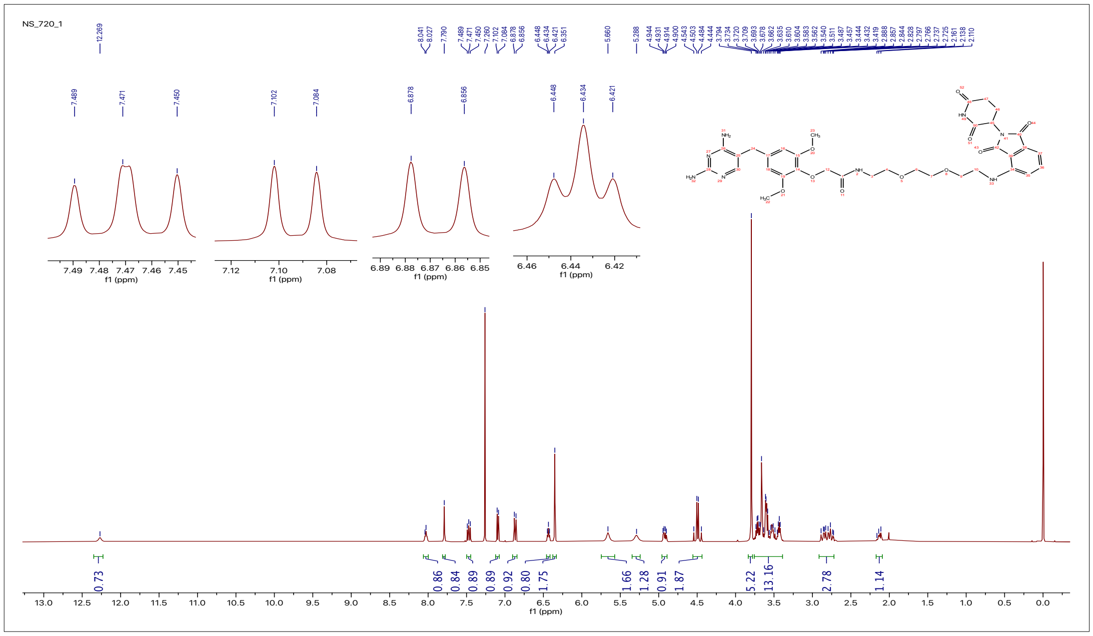

Supplementary Figure 12. ^{1}H NMR spectrum of compound **7a**.

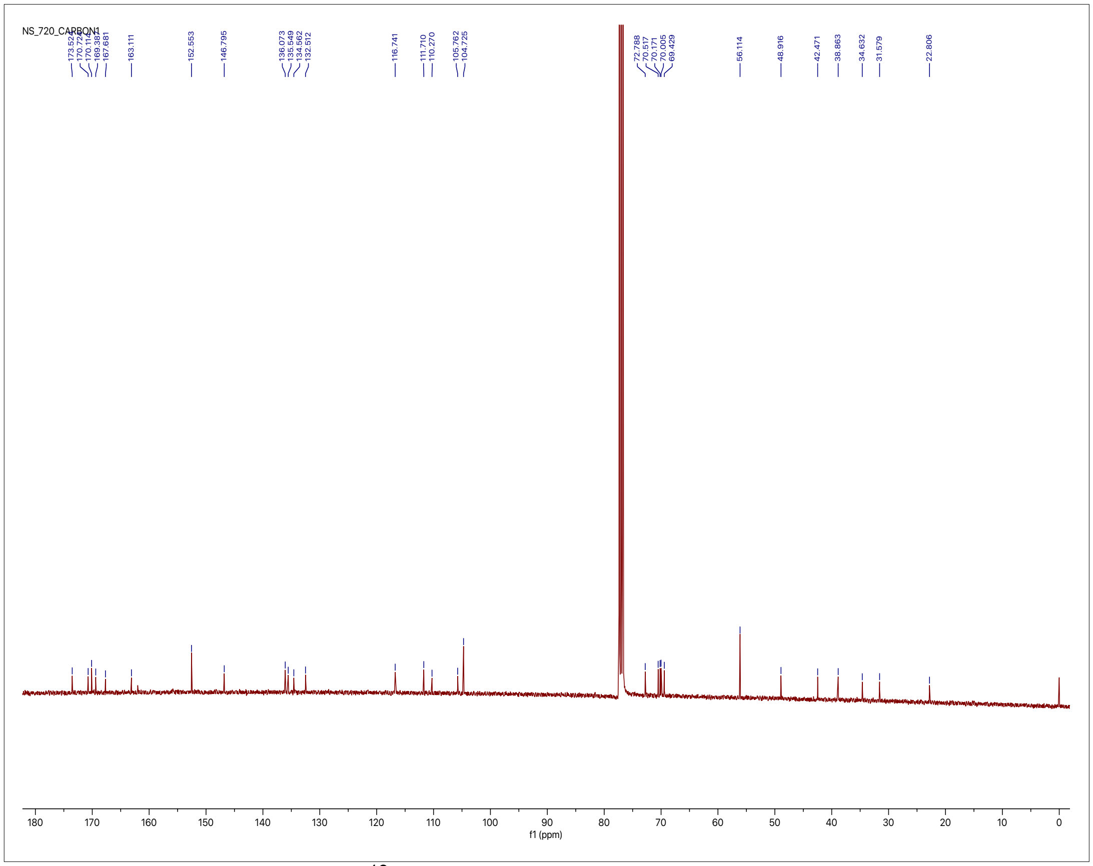

Supplementary Figure 13. ^{13}C NMR spectrum **7a**.

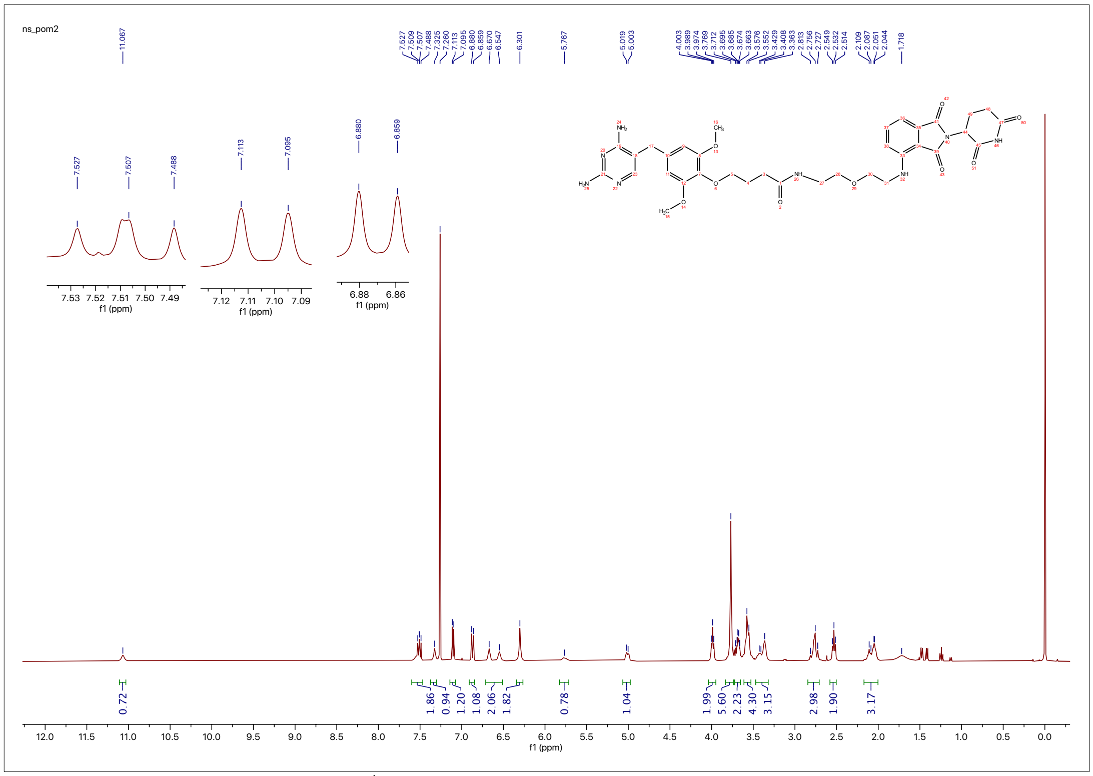

Supplementary Figure 14. ^{1}H NMR spectrum **7b**.

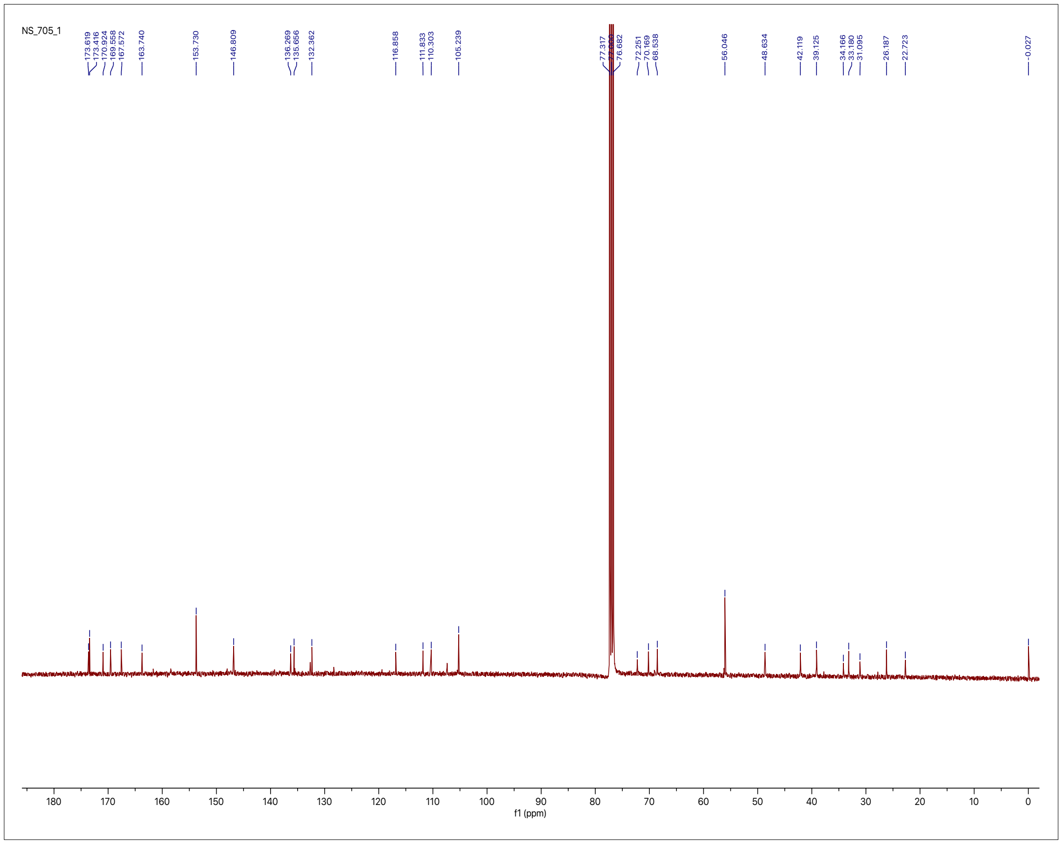

Supplementary Figure 15. ^{13}C NMR spectrum **7b**.

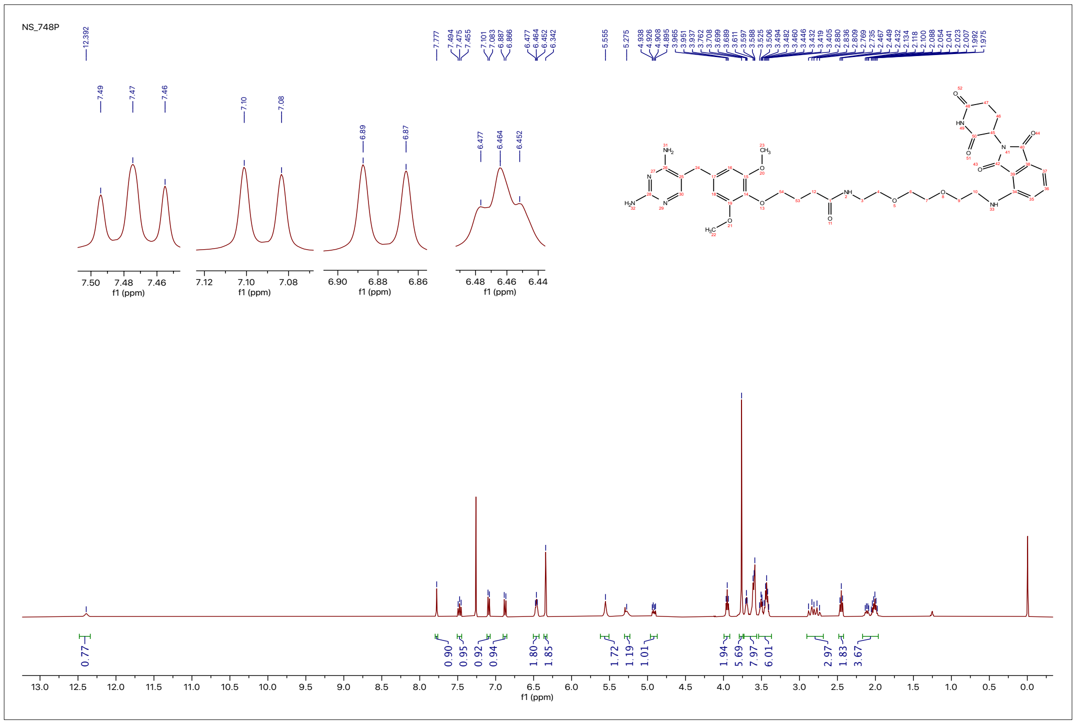

Supplementary Figure 16. ^{1}H NMR spectrum **7c**.

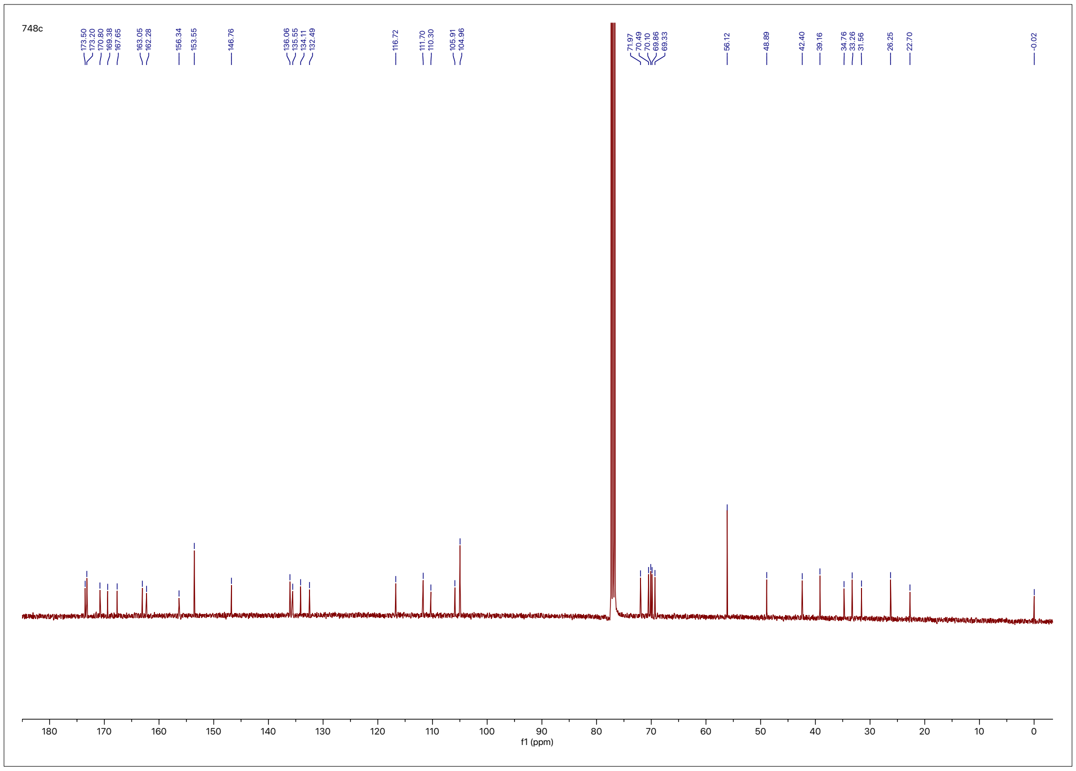

Supplementary Figure 17. ^{13}C NMR spectrum **7c**.

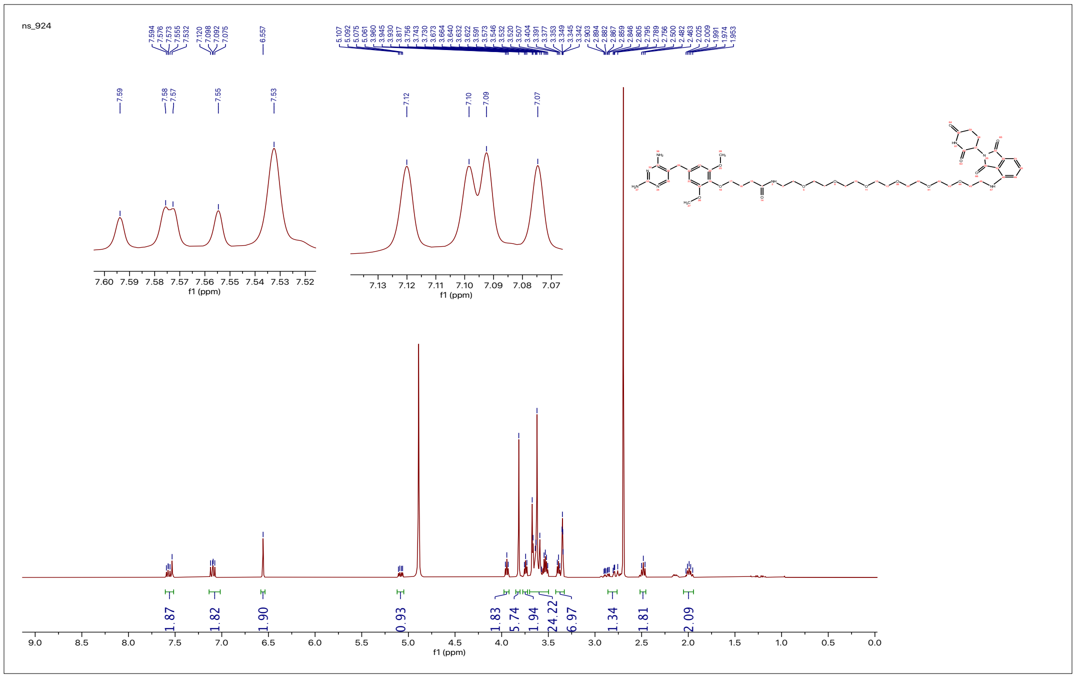

Supplementary Figure 18. ^{1}H NMR spectrum **7e**.

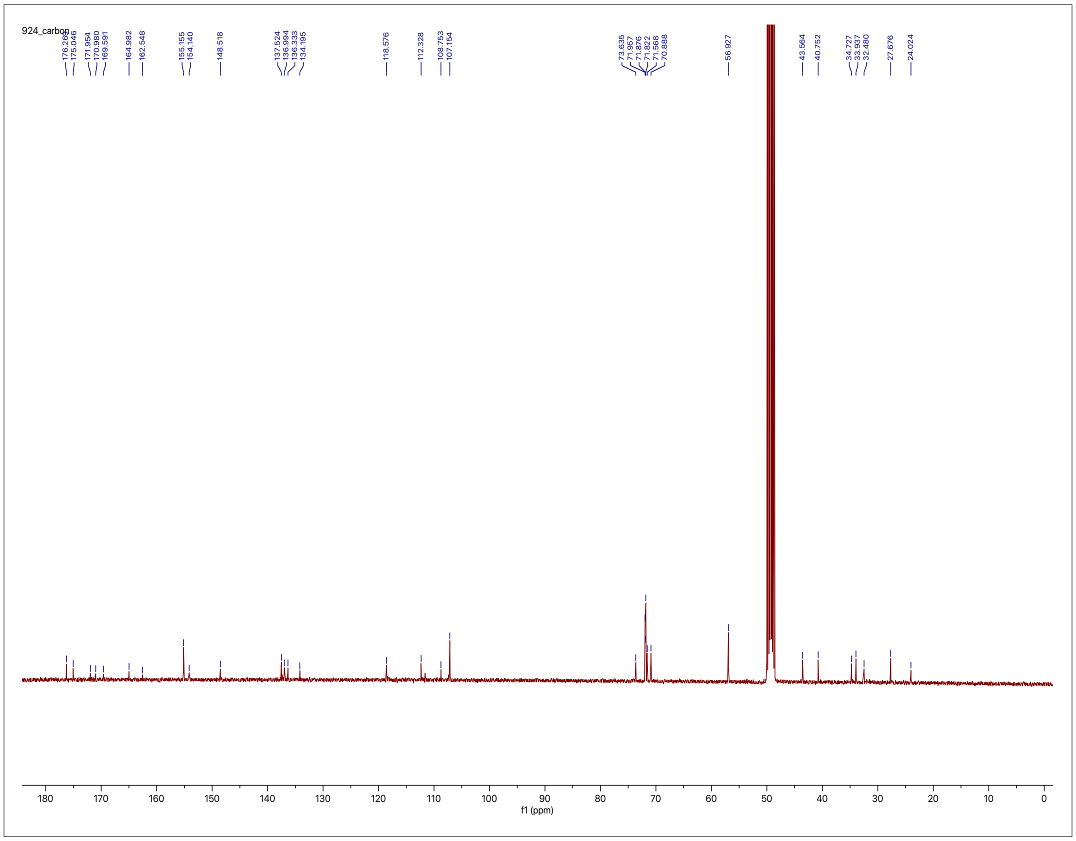

Supplementary Figure 19. ^{13}C NMR spectrum **7e**.
